# The SystHERs registry: an observational cohort study of treatment patterns and outcomes in patients with human epidermal growth factor receptor 2–positive metastatic breast cancer

**DOI:** 10.1186/1471-2407-14-307

**Published:** 2014-05-02

**Authors:** Debu Tripathy, Hope S Rugo, Peter A Kaufman, Sandra Swain, Joyce O’Shaughnessy, Mohammad Jahanzeb, Ginny Mason, Mary Beattie, Bongin Yoo, Catherine Lai, Anthony Masaquel, Sara Hurvitz

**Affiliations:** 1 Keck School of Medicine, University of Southern California, USC Norris Comprehensive Cancer Center, Los Angeles, CA, USA; 2 University of California San Francisco, Helen Diller Family Comprehensive Cancer Center, San Francisco, CA, USA; 3 Norris Cotton Cancer Center, Dartmouth-Hitchcock Medical Center, Lebanon NH, USA; 4 Washington Cancer Institute, Medstar Washington Hospital Center, Washington, DC, USA; 5 Charles A. Sammons Cancer Center, Texas Oncology, The US Oncology Network, Dallas, TX, USA; 6 University of Miami Sylvester Comprehensive Cancer Center Deerfield Campus, Deerfield Beach, FL, USA; 7 Inflammatory Breast Cancer Research Foundation, West Lafayette, IN, USA; 8 Genentech, Inc, South San Francisco, CA, USA; 9 University of California at Los Angeles Jonsson Comprehensive Cancer Center, Los Angeles, CA, USA

**Keywords:** Ado-trastuzumab emtansine, Human epidermal growth factor receptor 2, HER2, Metastatic breast cancer, Observational cohort study, Patient-reported outcome, Pertuzumab, Registry, SystHERs, Trastuzumab, Trastuzumab emtansine

## Abstract

**Background:**

Amplification of the *human epidermal growth factor receptor 2* (*HER2*) gene occurs in approximately 20% of invasive breast cancer cases and is associated with a more aggressive disease course than HER2-negative breast cancer. HER2-targeted therapies have altered the natural history of HER2-positive breast cancer, a trend that will likely further improve with the recent approval of new agents. A prospective, observational cohort study was designed and initiated to provide real-world insights into current treatment patterns, long-term survival, and patients’ experiences with initial and subsequent treatments for HER2-positive metastatic breast cancer (MBC).

**Methods/Design:**

The Systematic Therapies for HER2-positive Metastatic Breast Cancer Study (SystHERs) is a US-based prospective observational cohort study enrolling patients ≥18 years of age with recently diagnosed HER2-positive MBC not previously treated with systemic therapy in the metastatic setting. The primary objective of the study is to identify treatment patterns and clinical outcomes in recently diagnosed patients in a variety of practice settings. Secondary objectives include comparative efficacy, safety, and patient-reported outcomes (PROs). Healthcare resource utilization is an exploratory end point. Tumor tissue and blood sample collection is optional.

The SystHERs registry will enroll approximately 1000 patients over a 3-year period, after which the study will continue for ≥5 years, allowing for a maximum follow-up of 8 years. The treating physician will determine all care and the frequency of visits. PRO measures will be completed at study enrollment and every 90 days. Clinical data will be abstracted quarterly from patient records. The first patient was enrolled in June 2012, and preliminary descriptive data based on 25% to 30% of the final study population are expected at the end of 2013, and as of April 25, 2014, 386 patients are enrolled.

**Discussion:**

SystHERs is expected to provide in-depth data on demographic, clinicopathological, and treatment patterns and their associations with clinical outcomes, PROs, and healthcare resource utilization. Tumor tissue and DNA repositories will also be established for use in future translational research.

**Trial registration number:**

NCT01615068 (ClinicalTrials.gov identifier).

## Background

**Figure 1:**
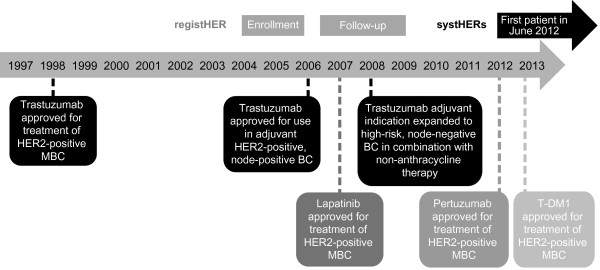
**Timeline of FDA approvals of HER2-targeted breast cancer therapies and conduct of the registHER and systHERs observational studies.** BC, breast cancer; FDA, US Food and Drug Administration; HER2, human epidermal growth factor receptor 2; MBC, metastatic breast cancer; OS, overall survival; SystHERs, Systematic Therapies for HER2-positive Metastatic Breast Cancer Study; T-DMI, trastuzumab emtansine.

The successful development of targeted agents for cancer therapy represents a major advance in personalized medicine, which strives to maximize therapeutic benefit while minimizing harmful side effects. In the field of breast cancer, human epidermal growth factor receptor 2 (HER2)–targeted therapies provide early examples of effective personalized medicine. The *HER2* gene is amplified in approximately 20% of invasive breast cancer cases [1,2], and this amplification is associated with an aggressive disease course [1,3]. To date, four HER2-targeted agents have received approval from the US Food and Drug Administration (FDA) for the treatment of patients with advanced HER2-positive breast cancer. Three of these agents have been approved in combination with chemotherapy: trastuzumab (a humanized monoclonal antibody that targets subdomain IV of HER2), lapatinib (a HER1/HER2 dual tyrosine kinase inhibitor), and, in combination with trastuzumab, pertuzumab (a humanized monoclonal antibody that targets domain II of HER2 [ie, the dimerization domain], thereby inhibiting receptor dimerization and subsequent signaling. Trastuzumab emtansine (T-DM1), an antibody-drug conjugate comprising the cytotoxic agent DM1 joined with a stable linker to trastuzumab, has been approved as a single-agent (Figure 1).

Prior to the advent of HER2-targeted therapy, the prognosis for patients with HER2-positive breast cancer was markedly worse than it was for patients with HER2-negative disease [1]. Since trastuzumab was approved for the treatment of metastatic breast cancer (MBC) by the FDA in 1998, patients with HER2-positive disease treated with HER2-targeted therapy now have a better prognosis than patients with HER2-negative disease receiving standard treatment [2].

The treatment paradigm for HER2-positive MBC continues to evolve as breast cancer is recognized as a heterogeneous disease with multiple phenotypes. Even within the same tumor, heterogeneity in gene expression can create challenges for identifying molecular targets for therapy, resulting in primary and acquired tumor resistance [4], which may explain, at least in part, the variable activity of targeted therapies [5,6]. On the basis of the results of a number of studies that have assessed therapies following progression on trastuzumab [7-12], it has been suggested that the ongoing blockade of HER2 leads to improved outcomes. This and new insights into growth factor receptor pathways continue to spur the development of novel HER2-targeted therapies. Future directions in the treatment of HER2-positive MBC may involve chemotherapy-free combined biologic treatment approaches in an effort to overcome tumor resistance and increase tolerability of treatment. This concept was demonstrated in a randomized trial with dual blockade using trastuzumab and lapatinib compared with lapatinib alone following exposure to trastuzumab [10], as well as in a phase II study showing activity of pertuzumab plus trastuzumab [13]. The CLEOPATRA study was a phase III randomized controlled trial that demonstrated improved progression-free survival (PFS) and overall survival (OS) in patients with metastatic HER2-positive breast cancer when pertuzumab was added to trastuzumab plus docetaxel [14,15]. The continued evaluation of potential biomarkers for predicting response to individual therapies will also be important [6,16].

The changing therapeutic landscape for patients with HER2-positive MBC provides multiple options and opportunities for these patients, but it also increases the complexity of clinical decision making. Physicians must take into account factors such as the optimal sequencing of treatments to achieve the best overall clinical and survival outcomes while also minimizing toxicity. Data from randomized clinical trials and treatment guidelines from agencies such as the National Comprehensive Cancer Network can help inform treatment decisions. However, since real-world patients with MBC often have very different characteristics than those enrolled in clinical trials, clinicians often must extrapolate best practices into therapeutic decisions in later lines of therapy for patients who maintain good performance status and are candidates for further therapy. Furthermore, as treatments evolve, it becomes difficult to conduct randomized controlled trials of all potential therapies and their combinations. Prospective observational studies represent an important complement to randomized controlled trials; they can offer insight into treatment patterns and long-term survival in much less selective patient populations. They can also provide data on comparative effectiveness of treatment regimens. In the United States, population-based data on MBC are available through several national programs and registries such as the Surveillance Epidemiology and End Results program and the Breast Cancer Family Registry. However, data from these sources often do not provide detailed information on evolving treatment regimens, practice patterns, therapeutic efficacy, or toxicities.

One US population–based prospective observational study of patients with HER2-positive breast cancer has been carried out. This multicenter registry study, registHER, enrolled 1023 patients with recently diagnosed HER2-positive MBC from December 2003 to February 2006, and followed patients until early 2009, or until death/discontinuation from the study. The objectives of registHER were to describe disease natural history and treatment patterns in patients with HER2-positive MBC, as well as the associations between specific treatments and patient outcomes [17]. Overall, 87% of patients were treated with trastuzumab in the first-line setting, and median survival from the time of MBC diagnosis was longer in patients treated with trastuzumab in their first-line treatment regimen than in those treated without first-line trastuzumab (35.9 versus 31.4 months) [18]. Another important finding from registHER was that in a multivariate model adjusting for prognostic factors (Eastern Cooperative Oncology Group performance status, hormone receptor status, site of first disease progression), the risk of death was decreased in patients who continued to receive trastuzumab after their first disease progression compared with those who did not (adjusted hazard ratio, 0.23; 95% confidence interval, 0.17–0.31) [18]. Furthermore, first-line treatment with a trastuzumab-containing regimen was associated with better OS and PFS in patients with HER2-positive/hormone receptor–positive MBC [19], improved PFS across all age groups [20], and longer OS in patients with central nervous system metastases [17,20]. Biological insights into the heterogeneity of this populations emerged when it was demonstrated that patients presenting with de novo compared with recurrent metastatic disease had a longer median survival [21]. Also, a latent class modeling approach revealed two biologically distinct groups of patients with widely diverging survival [22].

Since the closure of registHER enrollment in 2006, the treatment portfolio for HER2-positive MBC has changed significantly, with lapatinib, pertuzumab and T-DM1 approved for use in the metastatic setting (see Figure 1) [8,14,23]. Furthermore, since trastuzumab was first approved for adjuvant use in 2006, fewer patients treated in the adjuvant setting develop recurrent metastatic disease. Treatment with trastuzumab in the adjuvant setting may also affect the natural history and effect of subsequent therapies [6,24-26]. Set against the backdrop of evolving therapeutic options for HER2-positive MBC, the Systematic Therapies for HER2-Positive Metastatic Breast Cancer Study (SystHERs) has been established to address gaps in our knowledge about which treatments are chosen and administered to patients with HER2-positive MBC, the corresponding outcomes, and the patients’ perspectives on their experience over the course of their disease. The collected data will reflect currently available HER2-directed therapies in a real-world setting, as well as the costs associated with these therapies. The study has also been designed to collate pharmacovigilance data to allow for the detection and characterization of any new safety signals. Finally, a tissue repository will be created as part of this study, thereby providing a robust resource for future translational research.

## Methods/Design

**Figure 2:**
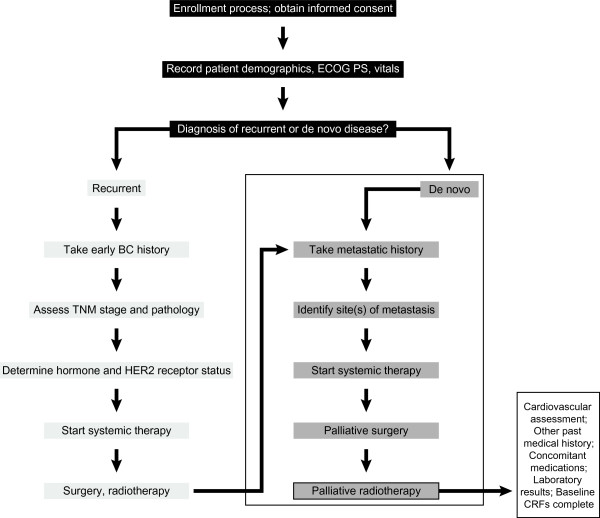
**Patient flow through the SystHERs registry at baseline.** BC, breast cancer; CRFs, case report form; de novo disease, previously undiagnosed metastatic breast cancer; ECOG PS, Eastern Cooperative Oncology Group performance status; HER2, human epidermal growth factor receptor 2; recurrent disease, first detection of metastases >90 days following histological diagnosis of early-stage breast cancer; SystHERs, Systematic Therapies for HER2-positive Metastatic Breast Cancer Study; TNM, tumor–node–metastasis.

SystHERs (ClinicalTrials.gov; NCT01615068) is a US-based, multicenter, prospective, observational cohort study. Its primary objective is to describe temporal trends in treatment patterns, the sequencing of treatments upon progression, and clinical outcomes (PFS, OS, key toxicities) in patients with HER2-positive MBC. Patients will undergo treatment and assessments in accordance with their treating physician’s standard practice; there is no study protocol–specified treatment regimen or evaluation schedule. The registry is enrolling patients within 6 months of their diagnosis of HER2-positive MBC. The first patient was recruited in June 2012, and data will be collected for up to 8 years. A total of 121 study sites are currently active. Patient flow through the study at screening/baseline is illustrated in Figure 2.

### Eligibility criteria

The registry is enrolling patients ≥18 years old with an initial diagnosis of HER2-positive MBC using physicians’ and institutional standards (based on the status of the primary tumor or biopsy of recurrence) within 6 months of the time of enrollment. Eligible patients must also have cancer-specific historical data points in their medical records. The only exclusion criteria for SystHERs are (1) any inability to provide informed consent and (2) diagnosis of HER2-positive MBC more than 6 months before enrollment. The inclusion criteria for SystHERs are intentionally broad in an effort to exclude as few patients as possible and thereby capture true population-based data.

### Ethical considerations

All participating patients must provide written informed consent and authorization to use their medical records; participation in translational research (ie, collection of blood and tissue for the DNA and tumor tissue repositories, respectively) is optional, and each requires separate consent. To maintain patient confidentiality, all patient-identifying information will be removed from tissue and blood samples prior to analysis, and it will therefore not be possible to link specific samples with their patient source. The study is being conducted in accordance with FDA regulations, the International Conference on Harmonization E6 Guidelines for Good Clinical Practice, the Declaration of Helsinki, and applicable local laws. Ethical approval has been obtained from all participating study sites in the form of written approval of the study protocol by the ethics committee or institutional review board at each site.

### Baseline evaluation and follow-up

Baseline patient and tumor characteristics, disease history, HER2 testing methodology, and previous cancer-related treatment data (including prior treatments for early-stage disease) are collected at enrollment. Subsequently, data on changes in cancer-related treatment, clinical outcomes, and adverse events (AEs) will be abstracted quarterly from patient charts, clinic notes, diagnostic tests, and laboratory findings. This includes tumor assessment (complete response, partial response, stable disease, progressive disease, unevaluable), method of tumor assessment, and sites of progression or new metastases. HER2, estrogen receptor, and progesterone receptor status is captured at both initial diagnosis and MBC recurrence. Patient-reported outcomes (PROs) are collected at study enrollment and every 90 days (Table 1).

**Table 1:** Timing of data assessments in the SystHERs registry

**Assessment**	**Time of data collection**		
	**Baseline (enrollment)**	**Follow-up (quarterly**^**a**^**)**	**Death or study discontinuation**
Informed consent	X		
Demographic data	X		
Functional/performance status	X	X	X
Cardiovascular risk factors	X		
Other relevant medical history	X		
Selected concomitant medications	X^b^	X	X
Selected laboratory tests	X	X	X
Breast cancer–specific cancer history	X^c^		
Prior cancer treatment history	X		
Healthcare resource utilization	X	X	X
Healthcare provider type	X		
Insurance coverage and provider	X	X	X
PROs^d^	X	X	X^e^
Tissue and blood samples^f^	X	X	
Disease status^g^		X	X
Chemotherapy treatment status		X	X
Interval surgical history		X	X
Interval radiotherapy history		X	X
Interval hormonal therapy history		X	X
Protocol-specified AEs^h^		X	X
Study discontinuation reason (including death)			X

^a^Patient data should be reported at the time of study termination.

^b^All breast cancer treatments (including neoadjuvant and adjuvant treatments and duration) and select concomitant treatments, including those for protocol-specified safety events, will be recorded.

^c^To include stage (at time of diagnosis), histology, estrogen receptor/progesterone receptor status, metastatic sites, staging diagnostic work-up, presence or absence of central nervous system metastases.

^d^PROs will be collected during clinic visits at approximately 90-day intervals (±15 days) and at frequent intervals to capture PRO data around disease progression (see Table 2 for details of PROs).

^e^An attempt will be made to collect PROs at study discontinuation.

^f^Consent is required for collection of blood and tissue samples. Blood and/or tissue sample(s) can be collected at any time while on study.

^g^Sites of disease progression and new metastatic sites will be recorded.

^h^These include all SAEs, selected AEs, AEs of special interest, and AEs/SAEs that lead to treatment discontinuation or modification. SAEs, pregnancies, and STIAMPs must be reported to Genentech within 24 hours of learning of the events.

AE, adverse event; PRO, patient-reported outcome; SAE, serious adverse event; STIAMP, suspected transmission of infectious agent by medicinal product, SystHERs, Systematic Therapies for HER2-Positive Metastatic Breast Cancer Study.

### Study end points

#### Primary end point: treatment patterns and sequencing

The primary end point is the distribution of patients receiving unique treatment regimens or a sequence of treatment regimens for HER2-positive MBC.

#### Secondary end points: clinical efficacy and safety

Secondary objectives of this study include comparing the efficacy and safety of HER2-targeted treatment regimens and evaluating the associations between patient characteristics, treatment variables and efficacy outcomes. Secondary clinical efficacy end points for this study are investigator-assessed PFS, OS, post-progression survival (measured once per patient, as the time from first investigator-assessed disease progression to death from any cause), time to treatment failure (ie, time from start of first-line HER2-targeted treatment to date of investigator-assessed disease progression, treatment discontinuation due to toxicity or death from any cause), and response rate (ie, proportion of patients with complete response or partial response based on their best overall response). Investigator-assessed disease progression may be based on clinical examination or radiographic or laboratory features, mirroring routine practice. All study assessments, including tumor response assessments, will be performed in accordance with institutional guidelines.

Safety end points are the incidences of serious AEs (SAEs), nonserious selected AEs, AEs/SAEs leading to treatment discontinuation or modification, and AEs of special interest (including pregnancy and suspected transmission of infectious agent by medicinal product [STIAMP]). SAEs are to be reported within 24 hours of the investigators’ awareness, while other AEs are to be graded using Common Terminology Criteria for Adverse Events (CTCAE) v4.0 and reported at quarterly data updates.

Selected AEs are cardiac dysfunction (≥1 of the following: left ventricular systolic dysfunction, congestive heart failure, cardiac arrest, cardiac ischemia/infarction), hepatotoxicity (severe drug-induced liver injury, nodular regenerative hyperplasia of the liver), thrombocytopenia associated with bleeding, infusion-related reactions/hypersensitivity, pulmonary events (pneumonitis, adult respiratory distress syndrome, interstitial lung disease), and CTCAE, v4.0, grade ≥3 febrile neutropenia [27].

#### Secondary end points: PROs

Another secondary objective of this study is to examine the association between HER2-targeted treatment regimens (and their sequencing) with PROs. Selection of PROs was based on a literature review, feedback from clinical experts, and the guidance document by Basch et al. regarding the incorporation of PROs in comparative effectiveness research in adult oncology [28]. Eight PRO measures are included that span a broad range of patient experiences such as symptom burden, ability to perform activities of daily living, and work productivity. Data from the following PRO assessments (Table 2) are collected in self-completed questionnaires at 90-day intervals (approximately) during clinic visits: Overall Health Related Quality-of-Life Question [Genentech, data on file], Functional Assessment of Cancer Therapy-Breast [29], alopecia patient assessment for patients with alopecia [Genentech, data on file], Patient Neurotoxicity Questionnaire [30], the MD Anderson Symptom Inventory Brain Tumor Module (only items related to cognitive function and interference with daily functioning) [31], Rotterdam Symptom checklist (only items relating to activities of daily living) [32], Work Productivity and Activity Impairment-Specific Health Problem questionnaire [33] (unemployed patients will answer two items), and the Health Care Survey (collects information on hospitalizations, emergency room visits, and out-of-pocket expenses) [Genentech, data on file]. Out-of-pocket expenses include co-payments, deductibles, transportation, and costs related to health care visits, as described previously [34]. It is estimated that patients will take 25 minutes to complete these quarterly PRO assessments. While it is preferred that this be performed at the physician’s office through an online interface using an iPad® (Apple Corp., Cupertino, CA), patients will also have the ability to complete the PROs at home using their own computer. Electronic capture of data will allow for rapid Web-based entry and will minimize difficulties inherent in the process of data transfer from paper to electronic databases.

**Table 2:** Key features of patient-reported outcome (PRO) scales used in the SystHERs registry

**PRO instrument**	**Key features**	**Frequency of administration**
Overall Health Related Quality of Life (HRQOL) question	Single question: “On a scale of 0–100, how would you rate your quality of life today?”	Every 90 days
Functional Assessment of Cancer Therapy-Breast (FACT-B) [29]	Self-report instrument designed to measure multidimensional QOL in patients with breast cancer	Every 90 days
Alopecia patient assessment	For use only in patients with alopecia; developed by Genentech	Every 90 days
Patient Neurotoxicity Questionnaire (PNQ) [30]	Assesses neurosensory and neuromotor symptoms on activities of daily living	Every 90 days
MD Anderson Symptom Inventory Brain Tumor Module (MDASI-BT) [31]	Only items related to cognitive functioning and interference with daily functioning will be administered	Every 90 days
Rotterdam Symptom Checklist [32]	Self-report instrument to measure QOL in cancer patients. Only items related to activities of daily living will be administered	Every 90 days
Work Productivity and Activity Impairment-Specific Health Problem (WPAI-SPH) questionnaire [33]	Patient-reported quantitative assessment of the amount of absenteeism, presenteeism, and daily activity impairment attributable to a specific health problem (breast cancer); unemployed patients will answer only 2 questions	Every 90 days
Health Care Survey	Quantifies hospitalizations, emergency room visits, and out-of-pocket expenses; developed by Genentech	Every 90 days

MBC, metastatic breast cancer; QOL, quality of life.

Patients are compensated every quarter for completing the PRO instruments in an effort to offset costs associated the extra time required of patients.

### Exploratory end points

Exploratory objectives of the study include health economic assessment and translational research. Health economic assessment will be captured by means of the Health Care Survey described in the previous section. For the exploratory objective of future translational research, the study protocol includes the optional collection of tumor tissue (including biopsies of metastatic sites if done as part of routine clinical care at the respective clinical site) for storage in a central tissue bank, as well as whole blood collection (at any time during the study) and subsequent DNA extraction for storage in a central DNA repository. Tissue samples may be in the form of paraffin-embedded primary tumor blocks from which cores may be taken to create tissue microarrays or up to 15 unstained microscope slides cut from those blocks. If whole tumor blocks cannot be donated to the repository, 6 to 8 cores can be taken from the sample for tissue microarrays and the reminder of the block returned to the site. Tissue and DNA samples may be stored and analyzed for up to 15 years after completion of the study. It is anticipated that this biospecimen repository will use high throughput technologies for gene expression analyses, genomic sequencing, identification of copy number/rearrangements, epigenetic status, and select protein analyses, permitting the identification of predictors of treatment response, long-term survival, and safety.

The patient data examined in this study will also enable the possible exploration of treatment compliance patterns. In particular, the source data include hospital records, clinical and office visit charts, and pharmacy dispensing records. Together, these sources will allow documentation of dose reductions of anticancer therapies, as well as the number of doses delivered. Collection of AE data and reasons for study discontinuation will enhance the interpretation of dose reductions and the duration and discontinuation of therapy.

### Recruitment plan

SystHERs has a planned enrollment of approximately 1000 patients and a recruitment period of approximately 3 years. Subsequent to full accrual, the study will continue for at least 5 years, allowing for a maximum follow-up period of 8 years.

To minimize selection bias, enrolling centers are being asked to systematically invite all eligible patients to participate in the study. Potential selection bias will be evaluated by gathering limited data (reason not enrolled, age range, race, disease-free interval, and performance status) on patients who meet all inclusion criteria but who are not enrolled.

### Electronic data capture

Patient clinical data and limited healthcare resource utilization data will be obtained from patients’ medical records via a Web-based password-protected/HIPAA compliant data collection system using electronic case report forms. The contract research organization will be responsible for the data management of this study, including quality checking the data, with Genentech performing oversight of the data management. System backups for data stored at Genentech and records retention for the study data will be consistent with Genentech’s standard procedures. PROs (including self-reported healthcare resource utilization data) will be collected on site approximately every 90 days throughout the study during clinic visits that are determined by the treating physician. However, if a treating physician does not schedule a quarterly visit, patients will have the opportunity to access the PROs at home via a Web interface to decrease the amount of missing data.

### Governance structure

The SystHERs steering committee provides scientific oversight for the study and is responsible for reviewing patient enrollment/withdrawal from the study, providing recommendations regarding changes in study conduct, reviewing results or interim analyses, conception of new analyses, and participating in drafting and publishing study reports. The steering committee comprises nine academic and community oncologists and two breast cancer advocates; a steering committee chair is elected from these members annually. The steering committee meets quarterly and operates under an approved charter, which specifies roles and responsibilities of committee members, as well as plans for the dissemination and publication of data from SystHERs. The steering committee charter also describes how decisions will be made, how study priorities will be set, and how plans will be executed.

### Statistical analysis

Continuous variables will be summarized using descriptive statistics and, where applicable, analysis of variance/model (*t*-test or F-test) or nonparametric testing (such as Wilcoxon’s rank sum test [35] or Kruskal-Wallis test [36]) will be used to test group differences. Categorical variables will be summarized by numbers and proportions, and, where applicable, chi-squared testing will be used to test group differences. The distribution of patients, as well as the duration of receiving unique treatment regimens or sequences of treatment regimens for HER2-positive MBC, will be summarized by each line of treatment.

Comparative effectiveness end points will be analyzed using standard statistical methods for cohort studies. Time-to-event end points will be summarized by the Kaplan-Meier method [37], and multivariate Cox regression analysis [38] will be performed to identify factors associated with these end points. Inverse probability weighted Kaplan-Meier estimates will be conducted as a sensitivity analysis for time-to-event end points. Potential confounders will be identified and controlled for, as appropriate, using analytical methods such as stratification, matching, multivariable regression modeling, and/or propensity score. A Cox proportional hazards model [38] with a time-dependent definition for treatment exposure will be used when survival bias is suspected. PRO end points will be summarized by quarterly study visit and will be analyzed using a mixed-effects model framework.

Incidence rates of SAEs, selected AEs, AEs causing treatment discontinuation/modification, and AEs of special interest (eg, pregnancies and STIAMP) will be summarized for all patients and by treatment regimen and treatment sequence in terms of the proportion of patients experiencing these outcomes and as incidence rates (events per 100 patient-years at risk). Exploratory analyses using logistic regression may be performed to identify risk factors for safety end points.

The planned sample size of 1000 patients was chosen to provide a sufficient number of patients to characterize treatments and measure overall trends by subgroups of common treatment regimens in a broad population. This sample size was determined to optimize the precision of measuring median survival outcomes based on assumed median OS and PFS times obtained from trastuzumab clinical trial data [39] and the registHER study. The sample size was also calculated to provide sufficient “exposed” and “control” patients to detect survival differences between treatment regimens, on the basis of currently available data on treatment regimen utilization and PFS and OS estimates obtained from trastuzumab clinical trial data [39] and the registHER study [Genentech, data on file].

## Discussion

The treatment options for patients with HER2-positive MBC have changed dramatically in the last decade, and real-world data are lacking on the natural history, treatment patterns, and outcomes in the era of second and third generation anti-HER2 therapies. SystHERs will provide data to address this need, and the inclusion of PROs and a biorepository will provide further insight. Lessons learned from registHER have informed the design of SystHERs, such as fewer sites (with more patients at each site) and a longer follow-up period (at least 5 years versus a 3-year median follow-up in registHER). registHER was not able to fully capture data on the patient population with survival times longer than 3 years; for example, median OS for patients <65 years of age who had received first-line trastuzumab was 40.4 months [20]. Understanding the characteristics of these patients with longer survival times is of keen interest.

Other important differences between SystHERs and registHER are the inclusion of a comprehensive panel of PROs and quantification of healthcare resource utilization. PROs capture information directly from the patient, without modification or interpretation by an investigator [40]. Collecting PROs empowers patients to report health-related outcomes associated with their treatment or illness. Completing PRO questionnaires on a regular basis reduces recall bias and allows a broader perspective of patient experience over time and may provide a more accurate assessment of patients’ symptoms. Longitudinal data on the impact of multiple successive treatment regimens on PROs are scarce. Building on the design of the PRO substudy in the VIRGO Metastatic Breast Cancer Registry, a US-based observational cohort study that followed more than 1200 women with primarily HER2-negative locally recurrent breast cancer or MBC [41], SystHERs is designed to provide a comprehensive PRO assessment with high patient adherence. This level of PRO assessment in an MBC registry study is unique to SystHERs and may provide valuable information regarding the experience of patients with HER2-positive breast cancer receiving treatment for metastatic disease and help patients and their clinicians make more informed treatment choices. The SystHERs protocol initially included the PRO-CTCAE (patient-reported outcomes version of the CTCAE) [42], but this instrument was eliminated during a protocol amendment since there are eight other PRO assessments and linking PRO-CTCAE results to specific treatments was judged to be infeasible in an observational registry with no mandated regimen or treatment schedule.

Another distinguishing feature of SystHERs is the establishment of a repository of tissue samples with the corresponding clinical outcomes data. This resource will permit future correlative studies with clinical outcomes to help tailor individualized treatment strategies in the future. In particular, it is anticipated that future studies will evaluate correlations of germline DNA characteristics and molecular events within the tumor with clinical outcomes, and identify inherited and somatic biomarkers that drive tumor progression, therapeutic responses, development of resistance, or predispositions for specific treatment toxicities.

### Methodological challenges

Enrolling 1000 patients with recently diagnosed HER2-positive MBC may be challenging since, due to the widespread use of adjuvant trastuzumab, fewer patients with HER2-positive early-stage breast cancer develop metastases. This may result in slow accrual of patients to the study. To address this, the planned enrollment period for the SystHERs registry is 3 years and will be expanded to include 146 sites with large patient populations (ie, those expected to enroll ≥10 patients in 3 years; approximately one patient per quarter) that are intended to be representative of the US population. SystHERs also has broad inclusion criteria and minimal exclusion criteria to enable the enrollment of a patient population reflective of the clinical practice setting. A potential limitation to the biobank is that submission of tissue and blood samples is optional and this may impact the exploratory analyses performed in the future.

The increasing complexity of therapeutic choices poses another challenge to the study design and analysis plan. The breadth of combinations of therapies, various sequencing patterns of therapies, and changes in available targeted therapies over the course of the study will make the grouping of types of treatments for analysis more difficult. This could potentially result in small patient numbers per group, reducing the statistical power to compare treatment outcomes. However, in this observational study, the primary objective is to describe the distribution of patients receiving unique treatment regimens or sequences of treatment regimens, rather than to perform direct comparisons between therapies. Comparative outcomes will be made along more general lines (eg, combination versus single-agent therapy with HER2-targeted agents and chemotherapy versus targeted therapy alone) and may inform future trials.

The large number of PRO measures being collected may reduce completion rates due to “questionnaire fatigue,” thereby increasing the amount of missing data. However, completion of the PRO scales will take just 25 minutes every quarter and some patients will not complete all questionnaires (eg, those without alopecia or not working), thus reducing the average time to complete the PROs. In addition, patients will be appropriately compensated for completing the questionnaire. These two factors should increase the likelihood of completion. In an effort to relax the underlying assumptions regarding missing data type, a likelihood-based mixed-effects model approach rather than a single imputation approach, such as last observation carried forward, will be used for the PRO analysis.

Investigators will not be required to use Response Evaluation Criteria in Solid Tumors (RECIST) to determine secondary efficacy end points. These end points will instead be assessed according to standard clinical practice at each institution. This approach has been adopted to capture the typical real-world experience of patients with MBC for whom the evaluation of tumor response may not be conducted in accordance with predefined criteria, such as the objective RECIST response criteria typically employed in oncology clinical trials. A drawback of this approach is the potential for variation between investigators and between study sites in how response is evaluated. However, investigators are encouraged to document their response assessment methods; thus, variations in assessment methodology can be investigated retrospectively. AEs will be reported in SystHERs in a similar manner to how they are reported in clinical trials. While offering a uniform approach between clinicians and across study centers, this approach may not best reflect the real-world experience of MBC patients in routine oncology practice. Additionally, patients enrolled in SystHERs may simultaneously be enrolled in a blinded randomized controlled trial; thus the study treatment received by the patients may not be known until after the trial is unblinded.

### Future directions

With a 3-year enrollment period and an additional 5 years of follow-up, SystHERs is well positioned to capture changes to the targeted treatment of HER2-positive MBC treatment resulting from ongoing clinical studies of emerging therapies and of combination treatment with existing therapies. Phase III trials are underway investigating the clinical impact of concomitant treatment with agents targeting multiple HER-family proteins, such as trastuzumab plus lapatinib and T-DM1 plus pertuzumab [43]. Investigations into combined HER-family blockade will increase as new HER2–HER4 multi-kinase inhibitors, such as afatinib and neratinib (which also inhibits EGFR), advance into late-stage trials [43]. There is also interest in combining HER2-targeted therapies with agents that inhibit signaling molecules downstream of HER-family receptors, such as PI3K, Akt, and mTOR. A phase III trial has demonstrated modest benefit for trastuzumab and vinorelbine plus the FDA-approved mTOR inhibitor everolimus in patients with trastuzumab-insensitive, HER2-positive MBC. [43] Early-stage trials seek to evaluate treatment with trastuzumab or lapatinib in combination with investigational inhibitors of PI3K or Akt [43]. By including clinical trial participants, SystHERs may provide early insight into the safety and efficacy of combination treatment with HER2-targeted therapies and some of these investigational agents.

Moreover, SystHERs has the potential to elucidate ongoing trends in patient selection for HER2-targeted therapy and to provide additional insight into the factors that predict clinical benefit from HER2-targeted therapy. SystHERs allows patients to donate tumor and blood samples for future study.

## Conclusion

The SystHERs registry aims to capture key characteristics of patients with HER2-positive MBC at or near to the time of their diagnosis and then follow them prospectively to gather information on the treatments they receive and the corresponding outcomes. This will allow quantitative, descriptive, and comparative analyses, which will evaluate associations between risk factors, treatments, and outcomes. Preliminary descriptive data based on initial enrollees is expected in late 2014. As a longitudinal observational cohort study, SystHERs will provide insight into the evolving treatment landscape of HER2-positive metastatic breast cancer.

## Abbreviations

AE: Adverse event; CTCAE: Common Terminology Criteria for Adverse Events; FDA: US Food and Drug Administration; HER: Human epidermal growth factor receptor; MBC: Metastatic breast cancer; OS: Overall survival; PFS: Progression-free survival; PRO: Patient-reported outcome; RECIST: Response Evaluation Criteria In Solid Tumors; SAE: Serious adverse event; STIAMP: Suspected transmission of infectious agent by medicinal product; SystHERs: Systematic Therapies for HER2-Positive Metastatic Breast Cancer Study; T-DM1: Trastuzumab emtansine.

## Competing interests

SystHERs is funded by Genentech, Inc., a member of the Roche group. DT is an uncompensated consultant for Genentech, Inc. HR has received research funding from Genentech, Inc., F. Hoffmann La-Roche Ltd., and GlaxoSmithKline. PAK has received research funding and honoraria from Genentech, Inc. and has served as a compensated and uncompensated consultant to Genentech, Inc. SS is an uncompensated consultant for Genentech, Inc. and her institution has received research funding from Genentech, Inc. JO is a consultant for Genentech, Inc. MJ has received research support from Genentech, Inc. and has served on advisory boards as a consultant/advisor for Genentech, Inc. GM has no competing interests. MB, BY, CL, and AM are employees of Genentech, Inc. and hold stock in F. Hoffmann La-Roche Ltd. SH is an uncompensated consultant for Genentech, Inc.

## Authors’ contributions

DT, HR, PAK, SS, JO, MJ, GM and SH are members of the SystHERs Steering Committee. MB is the medical monitor of SystHERs and CL is the medical science director. BY is the study statistician and AM is the patient-reported outcomes specialist. All authors contributed to the concept of this manuscript as well as to early and advanced drafts. All authors read and approved the final manuscript.

## Pre-publication history

The pre-publication history for this paper can be accessed here:

http://www.biomedcentral.com/1471-2407/14/307/prepub
